# Long-Term Trend of Liver Cancer Mortality in Serbia, 1991–2015: An Age-Period-Cohort and Joinpoint Regression Analysis

**DOI:** 10.3390/healthcare8030283

**Published:** 2020-08-21

**Authors:** Irena Ilic, Sandra Sipetic Grujicic, Jovan Grujicic, Djordje Radovanovic, Ivana Zivanovic Macuzic, Sanja Kocic, Milena Ilic

**Affiliations:** 1 Faculty of Medicine, University of Belgrade, 11000 Belgrade, Serbia; 2 Institute of Epidemiology, Faculty of Medicine, University of Belgrade, 11000 Belgrade, Serbia; sandra.sipetic-grujicic@med.bg.ac.rs; 3 Department of Biochemistry, Ave Maria University of Florida, Miami, FL 34142, USA; ssipetic@yahoo.com; 4 Faculty of Medicine, University Nis, 18000 Nis, Serbia; medicus_epidemiology@yahoo.com; 5 Department of Anatomy, Faculty of Medical Sciences, University of Kragujevac, 34000 Kragujevac, Serbia; ivanaanatom@yahoo.com; 6 Department of Social Medicine, Faculty of Medical Sciences, University of Kragujevac, 34000 Kragujevac, Serbia; kocicsanja@yahoo.com; 7 Department of Epidemiology, Faculty of Medical Sciences, University of Kragujevac, 34000 Kragujevac, Serbia; drmilenailic@yahoo.com

**Keywords:** liver cancer, mortality, trends, joinpoint regression analysis, age-period-cohort analysis

## Abstract

*Background and Objectives:* Trends of liver cancer mortality vary widely around the world. The purpose of this study was to assess the trend of liver cancer mortality in Serbia. *Material and Methods:* Descriptive epidemiological study design was used in this research. The age-standardized rates (ASRs, per 100,000) were calculated using the direct method, according to the World standard population. Temporal trends were assessed using the average annual percent change (AAPC) with 95% confidence interval (95% CI), according to joinpoint regression. An age-period-cohort analysis was used to evaluate the underlying factors for liver cancer mortality trends. *Results:* In Serbia from 1991 to 2015, over 11,000 men and nearly 8000 women died from liver cancer. The trend in liver cancer mortality significantly decreased both in men (AAPC = −1.3%; 95% CI = −1.7 to −0.9) and women (AAPC = −1.5%; 95% CI = −1.9 to −1.1). For liver cancer mortality, statistically significant cohort and period effects were observed in both genders. *Conclusions:* The downward trends in liver cancer mortality in Serbia are recorded during the past decades.

## 1. Introduction

Liver cancer is the fourth most common cancer in the world; in 2018, liver cancer caused almost 800,000 deaths for both sexes combined (about 8.2% of all cancer deaths globally) [1,2]. By sex, liver cancer is the second leading cause of cancer death in males (accounting for 10.2% of the total cancer deaths in men), while among women liver cancer ranks sixth for mortality (accounting for 5.6% of the total cancer deaths in women). Based on GLOBOCAN estimates in 2018, liver cancer is the first leading cause of cancer death in men in 20 countries (Mongolia, Lao People’s Democratic Republic and Thailand in Asia, and in African countries such as Egypt, Niger, Mauritania, Senegal, Ghana, etc.), while it is the leading cause of cancer death among women in three countries (Mongolia, Lao People’s Democratic Republic, Guatemala) [2]. Relative to number of deaths, the GLOBOCAN estimated that 50% of liver cancer deaths worldwide occur in China [1].

The highest mortality rates are observed mainly in settings with lower Human development index, that include several countries in Eastern and South-Eastern Asia (Thailand, Republic of Korea, Singapore) and in Eastern Europe (Romania, Moldova, Armenia) [2,3]. Rates of liver cancer mortality are two to three times higher among men than women in most regions of the world [4,5,6]. The estimated 5-year survival rates for all stages of liver and intrahepatic bile duct cancer combined are less than 20% [1,5,6,7].

In the last decades, liver cancer mortality trends in both genders have been increasing in countries that have experienced relatively low rates, such as the United States of America, Canada, United Kingdom, and most of Central and Northern European countries [1,4]. Liver cancer mortality trends are decreasing in countries that have had high rates of liver cancer in the past, such as China, Japan, and the Republic of Korea.

From the 1990s to today, Serbia has been facing socio-economic and political problems due to changes in the political and economic environment. Today, Serbia is an upper-middle income economy, has one of the oldest populations in Europe and high burden of non-communicable diseases, with a health system equal for every citizen regardless of their status [8]. Insight into long-term trends of liver cancer mortality can inform healthcare authorities of the potential effects of prevention measures, changes in risk factors and identify populations at higher risk, all of which can help guide future research and public health practices. The aim of this study is to explore the temporal trend in liver cancer mortality in Serbia.

## 2. Material and Methods

### 2.1. Data Source

Data on liver cancer (i.e., malignant neoplasms of liver and intrahepatic bile ducts) mortality (site code 155 revision 9 and code C22 revision 10 of the International Classification of Diseases (ICD) to classify death, injury and cause of death) were obtained from the Statistical Office of Serbia (unpublished data). In Serbia, from 1991 to 1996, data about the main cause of death are classified according to the ICD9, and since 1997 the data processing of mortality statistics has been based on the ICD10 [8]. In accordance with the legislation in Serbia, the medical certification of death is mandatory to be filled in for all deaths. The time and cause of death can only be determined by a medical doctor. The death certificate is issued by a health institution (for each person who was treated and died in that health institution), while a coroner (who is a doctor authorized by the municipality to determine the time and cause of death) issues a certificate for each person who died outside of the health care institution. According to the World Health Organization (WHO) guidelines, the definition of the underlying cause of death includes a disease or injury that has started a series of diseases or an injury that has triggered a series of disease states that directly led to death. The WHO assessed the quality of the official mortality statistics in Serbia as medium quality (the completeness is >90% and ill-defined causes of deaths appear on <10% of registrations) [9].

Study included the entire population of Serbia (about 7.6 million inhabitants in 1991 and about 7.2 million inhabitants in 2015), according to the 1991, 2002 and 2011 censuses, with estimates of the Statistical Office of the Republic of Serbia for all other years of the study period. During the study period, population in Serbia was characterized by the increasing retrograde demographic trends: reinforced the biological depopulation (increased mortality, reduce fertility, etc.), transition trends in the structure of the population (the rapid aging etc.), increased trends in spatial mobility (refugees, exiled and internally displaced persons, emigration of educated young people to foreign countries, etc.), with unfavorable trends in economy (unemployment, decrease in living standards, increase in poverty, etc.), with the negative circumstances that have preceded this (civil wars, disintegration of the country, international sanctions, the NATO bombing) [8]. In 1999, the number of refugees in Serbia was about 500,000. To date, most refugees have been included in the Serbian population and the refugee population data could not be set aside as a special contingent.

### 2.2. Statistics

Mortality of liver cancer was represented using crude rates, age-specific and age-standardized rates expressed per 100,000 persons. The age-standardized rates (ASRs) were calculated by methods of direct standardization using the World standard population.

Variations in liver cancer mortality rates over time were evaluated using the joinpoint regression analysis (Joinpoint regression software, Version 4.5.0.1, available through the Surveillance Research Program of the United States National Cancer Institute), according to the method proposed by Kim et al. [10]. Joinpoint regression analysis was used to identify point where a statistically significant change in the trend occurred over time. We used the Grid Search Method. The analysis was conducted with the minimum number of joinpoints (a zero joinpoint, representing a straight line), followed by tests for model fit with a maximum of 5 joinpoints. The Monte Carlo Permutation method for tests of significance was used. We selected a model where errors are assumed to have constant variance (homoscedasticity). Using the Monte Carlo Permutation technique with the number of 4499 randomly selected data sets, the numbers and locations of the joinpoints, with the best fitted models for liver cancer trend were estimated. Additionally, the program performed a permutation procedure for the test of parallelism. To estimate the magnitude and direction of the trends, the annual percent change (APC) and average annual percent change (AAPC) with 95% confidence interval (95% CI) in cancer mortality in the period 1991–2015 was calculated. Joinpoint results are not shown for the subgroups aged <45 years, because fewer than 5 cases of liver cancer deaths occurred in each of the quinquennium in any year.

Further on, an age-period-cohort analysis was conducted in order to evaluate the effects of age, period, and birth cohort on the observed temporal trends using the US NCI web-based statistical tool, according to the method proposed by Rosenberg et al. [11]. The age-period-cohort analysis used the liver cancer mortality data by consecutive 5-year age groups (45 to 49, …, and 80 to 84), and the 5-year intervals for calendar periods (1991 to 1995, 1996 to 2000, …, and 2011 to 2015) and birth cohorts (1911 to 1915, …, and 1966 to 1970). The central age group, time period, and birth cohort were defined as the reference categories and have effects set to zero. The estimable functions of the age-period-cohort analysis included period and cohort rate ratios, and local drifts with net drift: variations in mortality rates over time that influence all age groups simultaneously represent period effects; changes in mortality rates across groups of individuals with the same birth years represent cohort effects; local drifts represent annual percentage changes in mortality for each age group over time; net drift indicates the overall average annual percentage change in the mortality over the study period. The significance test was a 1-df Wald test. Considering that there have been very few cases in the age groups below 45 years or above 85 years and, consequently, mortality rates were unstable, we have omitted these age groups from the age-period-cohort analysis.

Both analyses, the joinpoint analysis and the age-period-cohort analysis, were performed by gender. In all analyses, a *p* value less than 0.05 was regarded as statistically significant.

### 2.3. Ethical Considerations

This study was approved by the Ethics Committee of the Faculty of Medical Sciences, University of Kragujevac (protocol: 01-4801).

## 3. Results

In Serbia from 1991 to 2015, over 11,000 men and approximately 8000 women died from liver cancer (Table 1). In men, the average annual age-standardized mortality rate was 6.9 per 100,000. The average annual age-standardized mortality rate was 3.7 per 100,000 in women.

**Figure 1:**
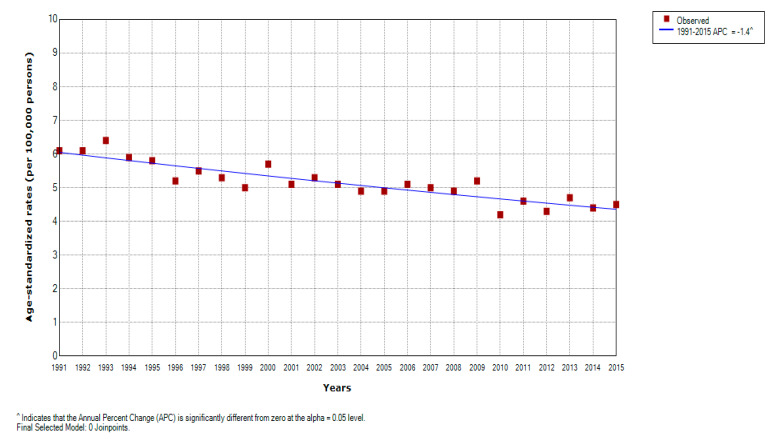
Joinpoint regression analysis of liver cancer mortality in Serbia, 1991–2015: All 0 Joinpoints. Legend: ^ Statistically significant trend; APC, Average Percentage Change.

**Figure 2:**
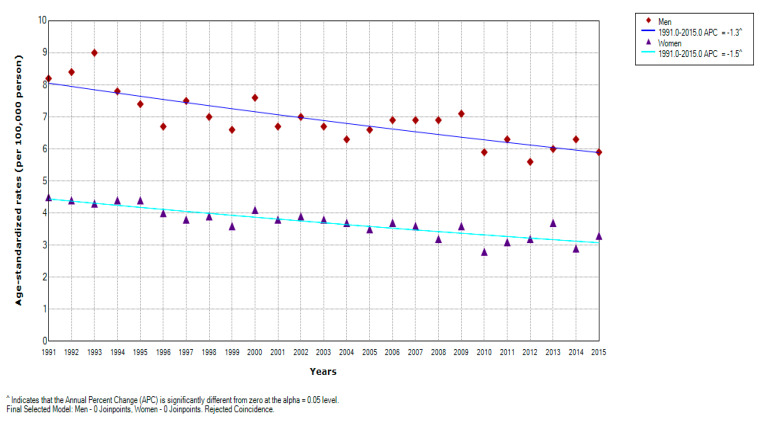
Joinpoint regression analysis of liver cancer mortality in Serbia, by gender, 1991–2015: Men: 0 Joinpoints vs. Women: 0 Joinpoints. Legend: ^ Statistically significant trend; APC, Average Percentage Change.

There was an overall significant decreasing trend for liver cancer mortality in Serbia over the entire observed period (by −1.4% yearly, 95% CI = −1.6 to −1.0) (Figure 1). Significantly decreasing trends for liver cancer mortality were observed during the entire study period in both genders: a significant decrease by −1.3% per year (95% CI = −1.7 to −0.9) in men and a significant decrease by −1.5% per year (95% CI = −1.9 to −1.1) in women; no one joinpoint was detected (Figure 2, Table 2). According to the comparability test, liver cancer mortality trends in men and women were parallel (final selected model: failed to reject parallelism: *p* = 0.403).

In Serbia, the liver cancer mortality rates increased with age in both genders, except in oldest age, among 85+ aged (Table 2). In men, liver cancer mortality trends were decreasing in age groups <80 years, although in those aged 50–54 and 75–79 the decrease was not significant. In women, the decrease in liver cancer mortality was significant for age groups 55–79, but non-significant downward trends were observed in younger age groups (45–49 and 50–54) and in older age group 80–84. There are no joinpoints, with one exception for 50–54-year-old women, in whom a significant decreasing trend in the period 1991 to 1998 was followed by a non-significant increase from 1998 to 2015.

**Figure 3:**
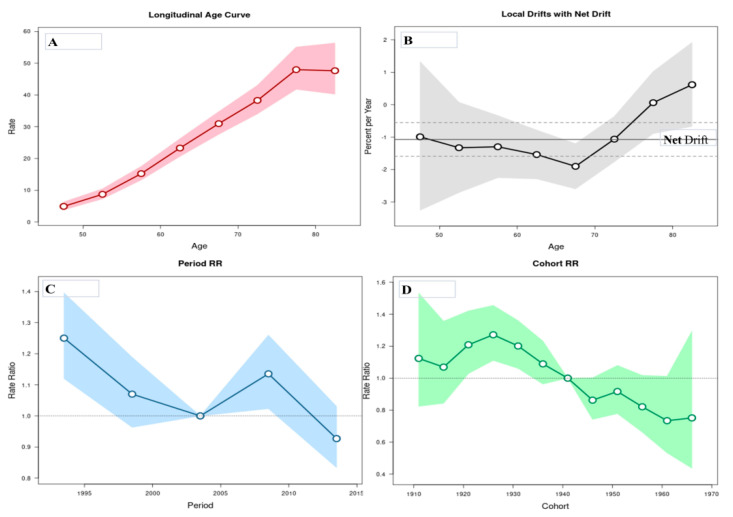
Liver cancer mortality rates in Serbia, 1991–2015: An age-period-cohort analysis. (**A**) Longitudinal age curve of liver cancer mortality rates (per 100,000 people) and 95% confidence intervals (the area colored in pink). (**B**) Local drift value: age group-specific annual percent change (%) and 95% confidence intervals (the area colored in grey), and net drift. (**C**) Period effects for the liver cancer mortality rates and 95% confidence intervals (the area colored in blue); RR—rate ratio. (**D**) Cohort effects for the liver cancer mortality rates and 95% confidence intervals (the area colored in *green*); RR—rate ratio.

The age-period-cohort analysis of mortality from liver cancer in Serbia is showed in Figure 3. The risk of death from the liver cancer increased with age until a peak at aged 75–80 years and showed a decline thereafter. The local drift values were under 0 in almost all age groups, with values around 0 at ages 75–84 years: the net drift was statistically significant (*p* = 0.0001), whereas the local drifts did not (*p* = 0.0554). The period effects have showed downward trends since 1991, with significantly elevated values at 2006–2010 years: period effect was statistically significant (*p* = 0.0001). The risk of death from liver cancer decreased for most of the birth cohorts, with only a few significant exceptions for the cohorts from 1921 to 1941: cohort effect was statistically significant (*p* = 0.0002).

The age-period-cohort analysis’ parameters for the liver cancer mortality in men and women aged 45–84 are displayed in Table 3. The risk of death from liver cancer increased with age in both genders (but until a peak in those aged 80 years, mortality rates decreased thereafter only in men): Wald test indicated statistically significant the net drift (*p* = 0.0001 in men, and *p* = 0.0008 in women), while the local drifts did not (*p* = 0.6908 in men, and *p* = 0.7573 in women). The period effect was statistically significant at the beginning of period in men and women, while the period effects remained relatively stable for the thereafter period in both genders. The risk of death from liver cancer in men elevated for birth cohorts from 1921 to 1940, while it declined in birth cohorts 1946–1960 in women: the cohort effects were statistically significant in both genders.

## 4. Discussion

This study demonstrated favorable long-term trends of liver cancer mortality in Serbia. There was a significantly decreasing trend for liver cancer mortality over the entire observed period in both genders; the statistically significant cohort and period effects were observed among men and women.

Mortality rates vary about 10-fold in both genders worldwide: the highest rates are found in men in Thailand (25.4 per 100,000) and women in Guatemala (11.3 per 100,000), while the lowest rates were recorded in men in Paraguay (2.6 per 100,000) and women in Iceland (1.0 per 100,000) [1]. In the European region (according to the latest available data), the highest rates were recorded in Armenia (13.1 per 100,000 in men and 5.9 per 100,000 in women), while the lowest rates were recorded in Ukraine (3.0 per 100,000 in men and 1.3 per 100,000 in women). Liver cancer mortality places Serbia among countries with intermediate rates in the European region, similar to Bulgaria, Romania, Georgia, and Switzerland [1].

Differences in the prevalence of risk factors, cancer prevention, diagnosis and treatment, in addition to variations in data quality worldwide could explain the noted significant between-country variations in mortality from liver cancer. [12]. High rates in Thailand and some other parts of Asia are related to high prevalence of liver fluke infection and chronic hepatitis B virus (HBV) infection (over 5%), consumption of food contaminated with aflatoxin [13]. The HBV infection is most often responsible for liver cancer in the Asia-Pacific region, while in Japan and Australia it is chronic hepatitis C virus (HCV) infection [14]. A systematic review of worldwide prevalence of chronic HBV infection found low prevalence in Serbia estimated to 0.48%, as well as in the United States of America (0.27%), Ukraine (1.45%) [15]. In contrast, Thailand (6.42%), China (5.49%), Niger (15.48%), Bulgaria (5.61%) had the highest HBV prevalence rates. However, exposure to other risk factors was most common in Western countries, including obesity, diabetes, alcohol consumption, and smoking [16,17]. Moreover, the WHO data indicated that prevalence of tobacco smoking among males was 48.7% in China and Mongolia, and 44.6% in Serbia, while in the highest prevalence in females was recorded in Serbia (39.7%) [18]. Similar to other countries [1], liver cancer mortality rates are approximately 2 times higher in men than in women in Serbia. Some of the differences for cancer mortality in men and women in Serbia could partly be due to the fact that a large percentage of men in Serbia traditionally drink on a daily basis (even six times more than women), while the number of daily smokers who are men (32.6%) is significantly higher compared to women (26.0%) [19].

Between 2006 and 2016, age standardized death rates for liver cancer decreased in countries with high/low–middle socio-demographic index (SDI), and increased in countries with low SDI [20]. In America, mortality rates increased from 2.8 to 6.5 per 100,000 between 1975 and 2015, predominantly due to hepatitis C infection in the 1960s and 1980s, obesity, alcohol and exposure of veterans to parasites [21]. A significant decline in AAPC for liver cancer mortality was noted during the 1991–2014 period in urban China in both sexes (−1.1% for men and −1.4% for women), with an initial increase during 1991–2007, followed by a decline [22]. Similar to other countries [22,23,24,25], the decline in liver cancer mortality in Serbia is associated with the prevalence of established risk factors, improved diagnostics (e.g., the use of ultrasound in the early detection of liver cancer), improvements in the diagnosis and treatment of cirrhosis, but also with the introduction and improvement of antiviral therapy for viral hepatitis (in Serbia since 2000) [8]. Additionally, the overall prevalence of HBV and HCV infection in Serbia significantly declined in past decades [8]. Age-period-cohort analysis in Korea showed that liver cancer mortality rates significantly decreased after the national vaccination program for HBV was implemented [24]. In Serbia, the HBV vaccination became mandatory in 1990s for persons from high risk groups, and in 2002 for all children at birth, with coverage of about 95% [8].

International differences in liver cancer mortality trends suggest differences in the distribution of various risk factors by sex and in different age groups and over time. In general, liver cancer mortality rates are increasing with age [1,4,6]. In urban and rural China, age effects had the most influence on liver cancer mortality [22]. Similar findings were noted in Taiwan [26], in Osaka, Japan [27], and in Korea [28]. In Serbia, the more unfavorable trends in liver cancer mortality in the oldest (especially men) can be related to the fact that the population in Serbia is becoming one of the oldest in the world, that they are not covered by the schedule of mandatory vaccination against hepatitis B, and that autopsies are performed less often in the oldest in order to determine the cause of death [8]. Similar to some findings [29], diverse liver cancer mortality trend in the oldest population in both genders in Serbia was likely related to possible inaccuracies in cause-of-death classification due to comorbidities in this population. Based on the National Health Survey in Serbia in 2013, in the 35–44 age group there was the largest percentage of smokers (47%), while the smallest percentage was in the 75–84 age group (9.1%) and among the eldest population of 85+ (4%) [19]. Results of the Survey showed that in 2013 in Serbia 4.6% of the population consume alcohol daily, with the largest percentage of daily drinkers in 65+ years aged [19]. Additional, the incidence of hepatitis B and hepatis C infection has remained almost unchanged in middle-aged and elderly in Serbia in the last decades [8].

According to the result of the APC analysis in China [22] and Taiwan [30], the cohort effects on liver cancer mortality mainly referred to HBV, HCV and aflatoxins, and, later, the implementation of hepatitis B vaccination. The cohort effects on liver cancer mortality were similar in both gender in Serbia, indicating that the males and females had the same exposures to certain risk factors in their lifetime. In the period between the two great world wars (birth cohorts from 1921 to 1940), the population of Serbia was characterized by a dominant representation of the rural population, illiteracy, poor sanitation of settlements, the dominance of infectious diseases in national pathology, expensive drugs, lack of hygienic control of water, bread and other foods [31]. Most children and pupils suffered from acute infections, and the high number of diseases was primarily a consequence of inadequate diet and lack of vitamins. A diverse liver cancer mortality trend in the oldest population in male and female birth cohorts from 1931 to 1940 in Serbia was likely related to possible inaccuracies in cause-of-death classification due to comorbidities in this population. After the second world war, with the improvements of living conditions and industrialization, the overall healthcare in Serbia has improved. Based on the National Health Survey in Serbia in 2013, a considerable decrease of the percentage of daily smokers was noted in 2006 in comparison with 2000 (from 33.0 to 26.2, respectively: from 40.6 to 30.7 in men, and from 26.1 to 22.6 in women) [19]. Additionally, in 2006, the average number of weekly alcoholic drinks amount significantly decreased compared to 2000 (from 8.3 to 6.4) [19]. Similar to other countries [32], most of HBV and HCV infections in Serbia occur in persons aged 30–39, with a slow decline in incidence after the age of 40, indicating that exposure to infection occurred mainly in young people (aged 20–29) [8]. However, some differences in prevalence of risk factors (such as infections, occupational exposures, etc.) among groups and over time are still unknown.

However, international comparisons of liver cancer mortality should be approached with caution due to underreporting, which can occur in low-income countries where advanced diagnostic techniques might be lacking, and because of overestimation due to misclassification of metastatic sites as primary cancer [19,29]. Further decline in liver cancer mortality could be enabled through control of tobacco and alcohol consumption, and hepatitis B vaccination, particularly among high-risk groups.

### Strengths and Limitations of the Study

The strength of this study is utilization of the national database, because these data provide the most comprehensive estimates of liver cancer mortality in Serbia to date. Additionally, liver cancer mortality trends were analyzed by both joinpoint regression and age-period-cohort analysis, which allowed evaluation of the underlying factors for liver cancer mortality trends.

Our study has several limitations. First, although the WHO assessed the registration of death data in Serbia as having medium quality [9], there is always a question of reliability and validity of death certification for cancers. Second, there are no data about liver cancer incidence for the entire Republic of Serbia, which could be used to explain the decline in mortality trends during the whole observed period. Third, the limitation of our study is that the mortality pattern could be confounded by a lack of separate data on liver cancer deaths among refugees in Serbia. Additionally, collinearity among age, period and cohort effects is an inherent limitation of the age-period-cohort analysis. Therefore, certain interpretations about liver cancer trends presented in our study still need further verification in future analytic epidemiological investigations.

## 5. Conclusions

The downward trends in liver cancer mortality in Serbia are recorded during the past decades. Observed trends are probably attributable to prevention efforts and changes in risk factors prevalence. Further decline in liver cancer mortality should be enabled through more comprehensive preventive measures, particularly among high risk groups, through continued and improved efforts in addressing risk factors such as smoking, alcohol consumption, obesity, and chronic viral hepatitis and also through reinforcing the ongoing HBV immunization efforts.

## Figures and Tables

**Table 1:** Liver cancer mortality in men and women in Serbia, 1991–2018; number of cases, crude rates and age standardized rate (ASR) per 100,000 persons (using World standard population).

Year	Men			Women		
	Number	Crude rate	ASR	Number	Crude Rate	ASR
1991	452	12.2	8.2	315	8.1	4.5
1992	483	13.0	8.4	314	8.1	4.4
1993	521	14.0	9.0	318	8.2	4.3
1994	459	12.3	7.8	333	8.5	4.4
1995	439	11.8	7.4	337	8.6	4.4
1996	402	10.8	6.7	314	8.1	4.0
1997	467	12.6	7.5	305	7.8	3.8
1998	437	11.9	7.0	310	8.0	3.9
1999	424	11.6	6.6	301	7.8	3.6
2000	487	13.3	7.6	336	8.7	4.1
2001	433	11.9	6.7	315	8.2	3.8
2002	461	12.6	7.0	332	8.6	3.9
2003	449	12.3	6.7	306	8.0	3.8
2004	420	11.6	6.3	336	8.8	3.7
2005	428	11.8	6.6	304	8.0	3.5
2006	471	13.1	6.9	333	8.7	3.7
2007	467	13.0	6.9	312	8.2	3.6
2008	462	12.9	6.9	285	7.5	3.2
2009	490	13,8	7.1	325	8.6	3.6
2010	412	11.6	5.9	269	7.2	2.8
2011	459	13.0	6.3	297	8.0	3.1
2012	410	11.7	5.6	312	8.4	3.2
2013	429	12.3	6.0	336	9.1	3.7
2014	455	13.1	6.3	279	7.6	2.9
2015	434	12.5	5.9	310	8.5	3.3
Overall	11,251	12.4	6.9	7834	8.2	3.7

**Table 2:** Joinpoint analysis: trends in age-specific liver cancer mortality rates (per 100,000) in men and women in Serbia, 1991–2015.

Age ^†^	Men		Women	
	Average Age-Specific Rates	AAPC (95% CI)	Average Age-Specific Rates	AAPC (95% CI)
45–49	5.2	−1.6 * (−3.0 to −0.2)	2.8	−0.1 (−2.3 to 2.1)
50–54	9.9	−1.1 (−2.4 to 0.3)	5.2	−1.2 ** (−3.0 to 0.6)
55–59	18.5	−0.9 (−2.0 to 0.2)	9.7	−2.0 * (−2.9 to −1.1)
60–64	32.3	−1.5 * (−2.1 to −0.7)	15.8	−2.4 * (−3.2 to −1.5)
65–69	46.7	−1.9 * (−2.7 to −1.2)	23.0	−2.4 * (−3.2 to −1.6)
70–74	58.4	−1.6 * (−2.3 to −0.9)	32.0	−1.1 * (−1.9 to −0.4)
75–79	71.6	−0.3 (−1.0 to 0.5)	40.7	−0.8 * (−1.5 to −0.1)
80–84	64.7	0.0 (−1.1 to 1.1)	43.1	−0.1 (−1.3 to 1.0)
85+	45.1	1.8 (−0.7 to 4.4)	34.6	−0.1 (−1.2 to 1.1)
All		−1.3 * (−1.7 to −0.9)		−1.5 * (−1.9 to −1.1)

Abbreviations: AAPC = Average Annual Percent Change; CI = Confidence Interval; * Statistically significant trend; ** One joinpoint was observed: Trend 1 (1991–1998): annual percent change (APC) (95% CI) = −12.5 * (−20.4 to −3.8); Trend 2 (1998–2015): APC (95% CI) = +2.1 (−0.4 to 4.7); ^†^ Joinpoint results are not shown for the subgroups aged <45 years for mortality, because fewer than 5 cases of liver cancer occurred in each of the quinquennium in any year.

**Table 3:** Age, period, and cohort effects on liver cancer mortality in Serbia, 1991–2015, by gender.

Group		Men		Women	
		Rate Ratio	95% CI	Rate Ratio	95% CI
Age					
	45–49	6.5	4.7–8.9	3.9	2.5–6.0
	50–54	11.3	9.0–14.2	6.6	4.9–8.9
	55–59	18.9	15.8–22.6	11.5	9.2–14.5
	60–64	30.8	26.4–35.9	17.0	14.0–20.6
	65–69	42.1	36.4–48.9	22.6	18.7–27.1
	70–74	49.0	42.0–57.1	28.6	23.8–34.4
	75–79	58.0	48.3–69.6	35.2	28.4–43.6
	80–84	50.7	40.1–63.9	36.2	28.2–46.4
Period					
	1991–1995	1.3	1.1–1.5	1.2	1.0–1.4
	1996–2000	1.1	0.9–1.2	1.1	0.9–1.3
	2001–2005	1.0	1.0–1.0	1.0	1.0–1.0
	2006–2010	1.0	0.9–1.2	0.9	0.8–1.1
	2011–2015	0.8	0.8–1.1	0.9	0.8–1.1
Cohort					
	1911–1915	1.3	0.8–2.0	1.3	0.8–2.0
	1916–1920	1.2	0.9–1.7	1.2	0.8–1.7
	1921–1925	1.3	1.1–1.6	1.2	1.0–1.6
	1926–1930	1.3	1.1–1.6	1.2	1.0–1.5
	1931–1935	1.2	1.0–1.4	1.1	0.9–1.4
	1936–1940	1.1	1.0–1.3	1.0	0.8–1.3
	1941–1945	1.0	1.0–1.0	1.0	1.0–1.0
	1946–1950	0.9	0.8–1.1	0.8	0.6–1.0
	1951–1955	0.9	0.8–1.2	0.7	0.6–1.0
	1956–1960	0.9	0.7–1.2	0.7	0.5–1.0
	1961–1965	0.7	0.4–1.0	0.8	0.5–1.4
	1966–1970	0.7	0.3–1.3	0.6	0.3–1.6
		Wald Chi-square tests for estimable functions, *p*-value			
Net drift		0.0001		0.0008	
All period rate ratios		0.0014		0.0194	
All cohort rate ratios		0.0173		0.0397	
All local drifts		0.6908		0.7573	

CI = confidence interval.

## References

[1] Bray F., Colombet M., Mery L., Piñeros M., Znaor A., Zanetti R., Ferlay J. (2017). Cancer Incidence in Five Continents, Vol. XI (electronic version). Lyon Int. Agency Res. Cancer. http://ci5.iarc.fr.

[2] Bray F., Ferlay J., Soerjomataram I., Siegel R.L., Torre L.A., Jemal A. (2018). Global cancer statistics 2018: GLOBOCAN estimates of incidence and mortality worldwide for 36 cancers in 185 countries. CA Cancer J. Clin..

[3] Siegel R.L., Miller K.D., Jemal A. (2016). Cancer statistics, 2016. CA Cancer J. Clin..

[4] Lin L., Yan L., Liu Y., Qu C., Ni J., Li H. (2020). The Burden and Trends of Primary Liver Cancer Caused by Specific Etiologies from 1990 to 2017 at the Global, Regional, National, Age, and Sex Level Results from the Global Burden of Disease Study 2017. Liver Cancer.

[5] Ferlay J., Soerjomataram I., Dikshit R., Eser S., Mathers C., Rebelo M., Bray F. (2015). Cancer incidence and mortality worldwide: Sources, methods and major patterns in GLOBOCAN 2012. Int. J. Cancer.

[6] Howlader N.N.A.K.M., Noone A.M., Krapcho M., Miller D., Bishop K., Altekruse S.F., Mariotto A. (2016). SEER Cancer Statistics Review, 1975–2013.

[7] Wang L., Xian Y., Yang Z. (2019). Comparison of 10 prognostic staging systems in patients with advanced hepatocellular carcinoma. J BUON.

[8] Statistical Office of the Republic of Serbia Demographic Yearbook in the Republic of Serbia (1991–2018). http://pod2.stat.gov.rs.

[9] Mathers C.D., Fat D.M., Inoue M., Rao C., Lopez A.D. (2005). Counting the dead and what they died from: An assessment of the global status of cause of death data. Bull. World Health Organ..

[10] Kim H.J., Fay M.P., Feuer E.J., Midthune D.N. (2000). Permutation tests for joinpoint regression with applications to cancer rates. Stat. Med..

[11] Rosenberg P.S., Check D.P., Anderson W.F. (2014). A web tool for age-period-cohort analysis of cancer incidence and mortality rates. Cancer Epidemiol. Biomark. Prev..

[12] Mittal S., El-Serag H.B. (2013). Epidemiology of hepatocellular carcinoma: Consider the population. J. Clin. Gastroenterol..

[13] Shin H.R., Oh J.K., Masuyer E., Curado M.P., Bouvard V., Fang Y.Y., Hong S.T. (2010). Epidemiology of cholangiocarcinoma: An update focusing on risk factors. Cancer Sci..

[14] Ko K.P., Shin A., Cho S., Park S.K., Yoo K.Y. (2018). Environmental contributions to gastrointestinal and liver cancer in the Asia–Pacific region. J. Gastroenterol. Hepatol..

[15] Schweitzer A., Horn J., Mikolajczyk R.T., Krause G., Ott J.J. (2015). Estimations of worldwide prevalence of chronic hepatitis B virus infection: A systematic review of data published between 1965 and 2013. Lancet.

[16] Cho L.Y., Yang J.J., Ko K.P., Park B., Shin A., Lim M.K., Yoo K.Y. (2011). Coinfection of hepatitis B and C viruses and risk of hepatocellular carcinoma: Systematic review and meta-analysis. Int. J. Cancer..

[17] Bosetti C., Turati F., La Vecchia C. (2014). Hepatocellular carcinoma epidemiology. Best Pract. Res. Clin. Gastroenterol..

[18] World Health Organization (2015). WHO Report on the Global Tobacco Epidemic, 2015.

[19] Fitzmaurice C., Akinyemiju T.F., Al Lami F.H., Alam T., Alizadeh-Navaei R., Allen C., Aremu O. (2018). Global, regional, and national cancer incidence, mortality, years of life lost, years lived with disability, and disability-adjusted life-years for 29 cancer groups, 1990 to 2016: A systematic analysis for the global burden of disease study. JAMA Oncol..

[20] Ministry of Health, Republic of Serbia (2014). National Health Survey, Serbia 2013.

[21] Siegel R.L., Jemal A., Wender R.C., Gansler T., Ma J., Brawley O.W. (2018). An assessment of progress in cancer control. CA Cancer J. Clin..

[22] Sun Y., Wang Y., Li M., Cheng K., Zhao X., Zheng Y., Wang L. (2018). Long-term trends of liver cancer mortality by gender in urban and rural areas in China: An age-period-cohort analysis. BMJ Open.

[23] Ziada D.H., El Sadany S., Soliman H., Abd-Elsalam S., Salama M., Hawash N., Selim A., Hamisa M., Elsabagh H.M. (2016). Prevalence of hepatocellular carcinoma in chronic hepatitis C patients in Mid Delta, Egypt: A single center study. J. Egypt Natl. Cancer Inst..

[24] Gwack J., Park S.K., Lee E.H., Park B., Choi Y., Yoo K.Y. (2011). Hepatitis B vaccination and liver cancer mortality reduction in Korean children and adolescents. Asian Pac. J. Cancer Prev..

[25] Ribes J., Clèries R., Borràs J., Galceran J., Bosch F.X. (2004). Time trends in incidence and mortality for chronic liver disease and liver cancer in the interval 1980–1997 in Catalonia, Spain. Eur. J. Gastroenterol. Hepatol..

[26] Su S.Y., Lee W.C. (2019). Mortality trends of liver diseases from 1981 to 2016 and the projection to 2035 in Taiwan: An age-period-cohort analysis. Liver Int..

[27] Ito Y., Ioka A., Nakayama T., Tsukuma H., Nakamura T. (2011). Comparison of trends in cancer incidence and mortality in Osaka, Japan, using an age-period-cohort model. Asian Pac. J. Cancer Prev..

[28] Park J., Jee Y.H. (2015). Age-Period-Cohort Analysis of Liver Cancer Mortality in Korea. Asian Pac. J. Cancer Prev..

[29] Sharp G.B., Cologne J.B., Fukuhara T., Itakura H., Yamamoto M., Tokuoka S. (2001). Temporal changes in liver cancer incidence rates in Japan: Accounting for death certificate inaccuracies and improving diagnostic techniques. Int. J. Cancer.

[30] Lee L.T., Huang H.Y., Huang K.C., Chen C.Y., Lee W.C. (2009). Age-period-cohort analysis of hepatocellular carcinoma mortality in Taiwan, 1976–2005. Ann. Epidemiol..

[31] Cirkovic S. (2004). The Serbs.

[32] Razavi H., Waked I., Sarrazin C., Myers R.P., Idilman R., Calinas F., Vogel W., Mendes Correa M.C., Hezode C., Lazaro P. (2014). The present and future disease burden of hepatitis C virus (HCV) infection with today’s treatment paradigm. J. Viral Hepat..

